# A Case Report on a Fractured Ceramic Bearing Surface Following Total Hip Replacement and a Short Review on the Mechanisms of Liner Fracture

**DOI:** 10.3390/reports7040117

**Published:** 2024-12-19

**Authors:** Calin Stefan, Cristian Moldovan, Liviu Marsavina, Mihai Hurmuz, Iuliana Stefan

**Affiliations:** 1 Department of Mechanics and Strength of Materials, Politehnica University Timisoara, Blvd. M. Viteazu, Nr. 1, 300222 Timisoara, Romania; 2 Orthopedics Unit, “Dr. Victor Popescu” Emergency Military Hospital, Gheorghe Lazăr Street 7, 300080 Timisoara, Romania; 3 Department XV, Discipline of Orthopedics, “Victor Babes” University of Medicine and Pharmacy Timisoara, Eftimie Murgu Square Nr. 2, 300041 Timisoara, Romania

**Keywords:** hip, implant, liner, fracture, ceramic, mechanical causes

## Abstract

**Background and Clinical Significance:** Since their first introduction in the early 1950s, hip prostheses implants are becoming increasingly reliable; nevertheless, failures can still happen. The focus of this paper is to present a case study on a catastrophic fractured hip prosthesis liner that consequently led to revision surgery and the replacement of the destroyed implant. **Case Presentation:** The patient was diagnosed with Hodgkin’s lymphoma and had Total Hip Arthroplasty on both legs, but only the right side needed revision due to a fracture in the liner. The patient’s symptoms were a squeaking sound, functional impairment, and pain, and an X-ray showed the extent of the damage. We also present a short review on the mechanisms of liner fracture, focusing strictly on the mechanical aspects of failure. **Conclusions:** Hip prosthesis implants are not immune to failure. This case highlights the importance of an interdisciplinary approach and emphasizes the need for vigilant postoperative monitoring and the development of predictive tools.

## 1. Introduction and Clinical Significance

**Figure 1:**
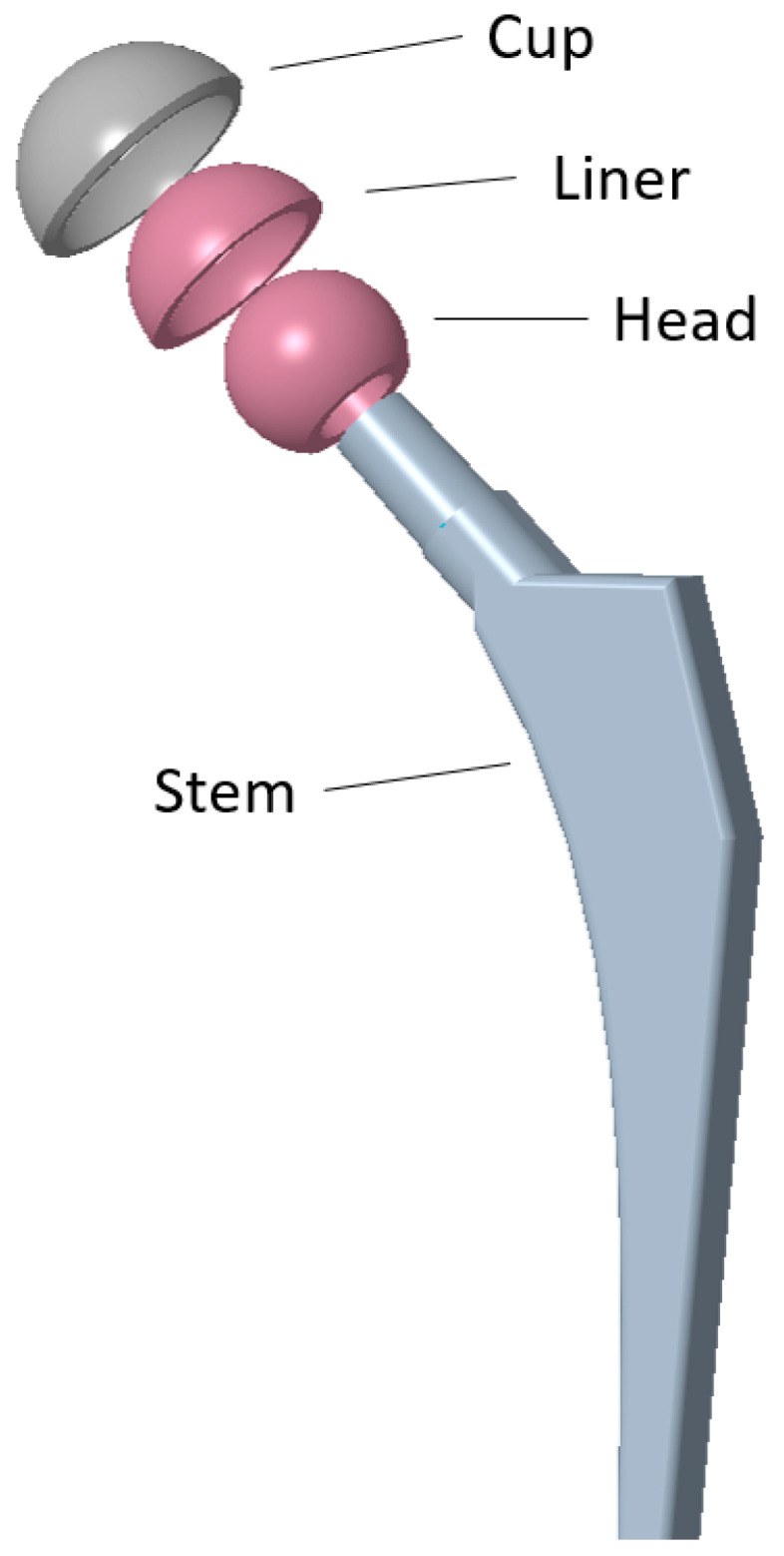
Components of a hip implant.

Total Hip Arthroplasty (THA) is a medical procedure in which a damaged hip joint is replaced with an implant in order to restore functionality and release pain in the patient. The term total (from Total Hip Arthroplasty) comes from the fact that both femoral and acetabular parts of the joint are replaced by an implant. A schematic of the implant is presented in Figure 1 [1], where the constitutive elements are pointed out. From these elements, we are especially interested in the liner, since in our case this element fractured, leading to revision surgery and the replacement of the damaged implant.

Historically, there has been an evolution of THA techniques, but the procedure that is adopted nowadays is still used, with minor modifications, since it was developed in 1950–1960 by Sir John Charnley [2]; he also introduced the concept of Low-Friction Arthroplasty. His first prosthesis used a polytetrafluoroethylene (PTFE or Teflon) cup, a 22.2 mm diameter head, and a stainless-steel stem, but PTFE was unsuitable, as it led to inflammatory reactions caused by wear and tear of the material. To solve these problems, Sir John Charnley adopted other materials, such as high-density polyethylene (HDPE), and ultra-high-molecular-weight polyethylene (UHMWPE). Using these material couplings, wear effects were reduced due to their smaller contacting surface and hard-on-soft coupling [3].

Modern implants use various constitutive materials. Of interest for us are the materials used to manufacture the implant head and liner, producing the spherical joint or bearing that mimics the natural hip joint. Many combinations of materials can be used, and these can be grouped into a few categories, listed as follows:

MoP—Metal head on polyethylene liner or cup (i.e., ultra-high-molecular-weight polyethylene—UHMWPE);

MoM—Metal on metal, generally a CoCrMo alloy;

CoC—Ceramic on ceramic, generally alumina, zirconia-toughened alumina ceramic (ZTA), or yttria-stabilized zirconia (YSZ);

CoP—Ceramic on polyethylene.

It is worth underlining that MoM implants have not been recommended for use ever since 2010, when the United States Food and Drug Administration (FDA) issued a warning against these kind of implants; apparently, the wear of the implant and the release of cobalt or chromium ions lead to a reaction that destroys the soft tissues surrounding the joint. Also, there is evidence that the ions entered the bloodstream and the cerebral spinal fluid in some patients. The FDA also recommends that patients should be monitored for systemic effects, particularly cardiovascular, neurological, renal, and thyroid signs and symptoms [4,5,6].

The head and liner of the implant studied in this paper is CoC and belongs to the product line BIOLOX Delta. In the literature, there are only a few documented cases of liner fracture; a review report from 2018 by [7] presents only six cases. The cases presented in [7] are extracted from [8,9,10,11], with conclusions on the cause of fracture as misalignment between cup and liner, external impact, and even one with an unknown/unidentified cause. In [10], the authors state that the probable causes of such fractures are manufacturing production failure and edge loading based on cup inclination, but, in their patient, inacceptable range of motion, failure of the locking mechanism during implantation insertion, or cracking were also possible causes of fracture. In paper [12], the authors present an internal hospital report for a period of 10 years after surgery on an alumina head from a Delta liner Total Hip Arthroplasty, where, from a population of 85 patients that had THA, only one case had revision surgery because of a fractured liner.

In [13], the authors present a case report where a patient had revision surgery due to the failure of the ceramic head, and in [7,14] the authors present a literature review on possible causes for implant failure.

The second chapter of this paper will present the case report of a patient who underwent revision surgery after a fractured ceramic liner. The third chapter presents a systematic approach on the mechanical causes for liner fracture, taking into consideration the lifecycle of an implant, namely, insertion, exploitation, and some additional case reports in the literature referring to cases of failure. The conclusion of the paper is that no clear cause for implant liner failure could be determined.

## 2. Case Presentation

The case report presented in this paper refers to a patient born in 1978, with a height of 181 cm and weight of 75 kg, having a body mass index (BMI) of 22.89 and placing him in the normal weight distribution.

The patient was diagnosed in July 2007 with Hodgkin’s lymphoma following a histological examination. Immediately after the diagnosis, he began treatment with ABVD polychemotherapy, administered in seven cycles until February 2008, followed by radiotherapy. The treatment results indicated partial remission.

In October 2015, Hodgkin’s lymphoma relapsed, requiring further treatment with BEACOPP chemotherapy, which again produced positive results. The amount of methylprednisolone used was 40 mg/day during days 1–14.

In February 2018, the patient was diagnosed with systemic avascular necrosis and bilateral aseptic necrosis of the femoral head, secondary to previous chemotherapy and radiotherapy treatments.

In November 2018, the patient was diagnosed with tricuspid insufficiency and moderate mitral insufficiency. As a result, surgical intervention was performed to implant a pacemaker and replace the mitral valve with a mechanical valve prosthesis (SJM Masters, 29 mm). The functionality of the tricuspid valve was also restored, and the surgery had a positive outcome.

In April 2019, the patient was admitted to the orthopedic ward, where a Total Hip Arthroplasty of the right hip was performed using uncemented ceramic–ceramic endoprosthesis. The new composite ceramic reduced the risk of fracture of the femoral head to 0.002%, according to [15,16]. The improved tribology means that CoC bearings are an excellent choice for young patients.

The surgical procedure followed this protocol:

• Lateral Hardinge approach of approximately 12 cm;
• Capsulotomy in a reverse “T” shape;
• Dislocation of the femoral head;
• Osteotomy of the femoral head;
• Excision of periarticular soft tissues;
• Preparation of the acetabulum with progressive rasps until 55 mm, followed by impaction of the acetabular component (Shell 56 mm Continuum Cluster holes Porous, Zimmer), and insertion of a Biolox Delta 36 mm ceramic insert;
• Preparation of the femoral canal;
• Preparation of the canal with progressive rasps, followed by impaction of the stem (Taperloc Stem No. 9, Biomet) and placement of the final standard 36 mm Biolox Delta femoral head;
• Reduction of the dislocation;
• Lavage.

**Figure 2:**
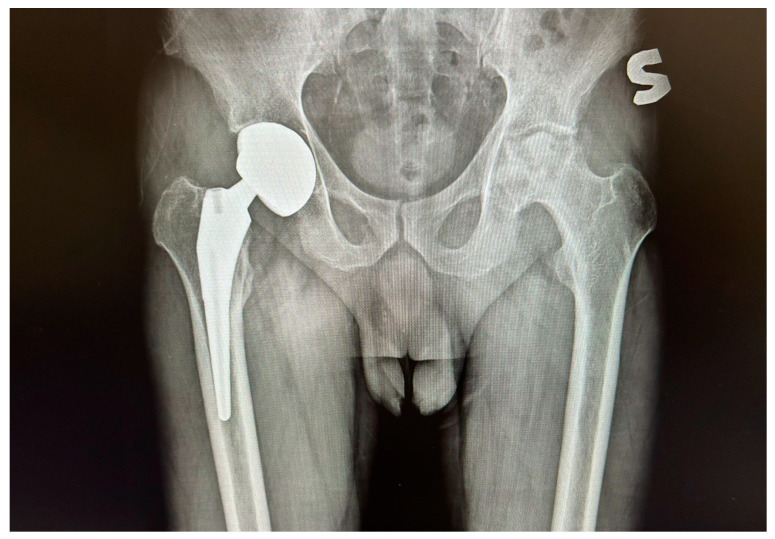
X-ray showing right implant after first THA.

The X-ray after the first THA (Total Hip Arthroplasty) procedure on the right side is shown in Figure 2.

In March 2020, the patient returned for Total Hip Arthroplasty of the left hip with uncemented ceramic–polyethylene endoprosthesis. During hospitalization, the patient complained of pain and partial functional impairment in the right hip joint, which had been operated on in the previous year. Clinical examination revealed a muscle strength deficit in the lower limb, including the quadriceps, gluteus maximus, abductors, and iliopsoas, along with a reduced range of motion in flexion, extension, and abduction of the hip.

The clinical examination was sufficient and concluded that revision surgery was necessary, without the necessity for an X-ray. Additionally, follow-ups were conducted at 3 and 6 months, and no signs of component failure were noted.

**Figure 3:**
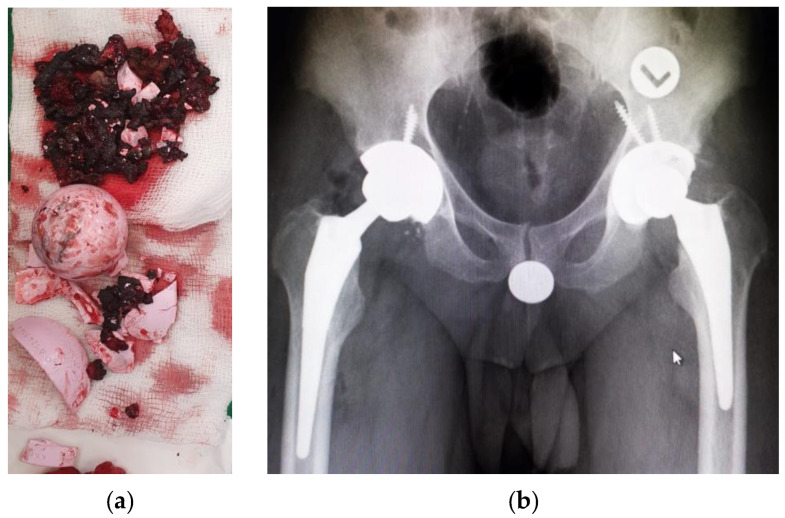
(**a**) Fractured ceramic liner and extracted debris during surgery; (**b**) X-ray showing right implant after the second THA.

In May 2020, surgical revision of the Total Hip Arthroplasty on the right side was performed, using uncemented ceramic–ceramic endoprosthesis. A hip X-ray after the second THA is presented in Figure 3b.

**Figure 4:**
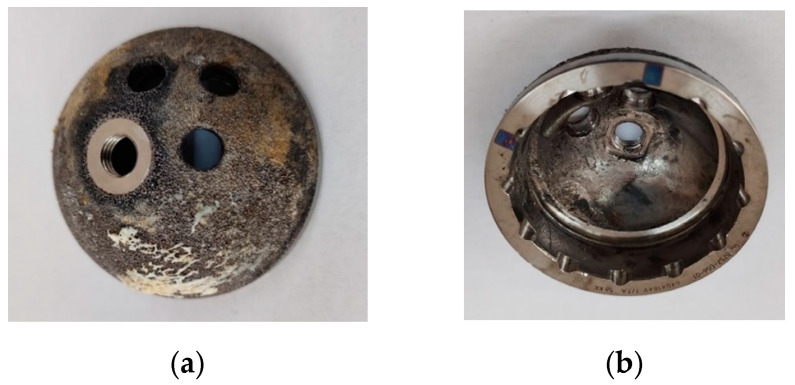
Implant cup component (**a**) exterior and (**b**) interior.

Figure 4 shows the cup component, with both interior and exterior surfaces; on the interior surface, wear can be observed due to the fragmentation of the liner component.

**Figure 5:**
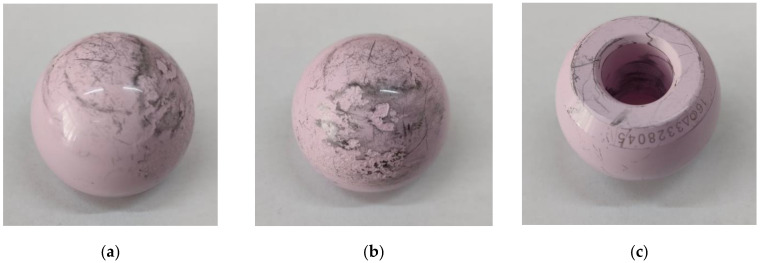
Implant head component (**a**) external surface view 1; (**b**) external surface view 2; (**c**) internal surface.

The implant head also shows severe degradation, as shown in Figure 5; one can also observe microchipping of the head’s external surface due to interaction with the fractured fragments of the ceramic liner.

It is also important to note that there was total revision of the stem, cup, liner, and head performed.

The postoperative evolution was favorable, and the patient was discharged to begin an intensive 6-month rehabilitation program. Although there were initial difficulties in regaining mobility and muscle strength, significant progress was made, and one year after the surgery, the patient was able to perform most daily activities without assistance.

Currently, four years after the last intervention, the patient has resumed daily activities, including professional work. His general condition is good, with no signs of recurrence or postoperative complications.

## 3. Discussion

This chapter presents a systematic approach on theoretically possible mechanical causes for liner fracture, taking into consideration the lifecycle of an implant, namely manufacture, insertion, and exploitation.

The fracture of the ceramic implant, such as the ceramic implant liner used in hip prostheses, is a rare event, but it can be caused by several factors. Here are some of the possible causes.

### 3.1. Manufacturing

During this stage, we consider defects that can appear during the manufacturing process.

#### 3.1.1. Material Faults

The quality of the ceramic material used in implants is crucial for long-term success. Any defect that occurs during the production process, such as microscopic pores or impurity inclusions, can weaken the structural integrity of the implant. These imperfections can arise from insufficient quality control of the raw materials or errors during the manufacturing process, such as improper sintering temperatures for the ceramic. Defective material will be more susceptible to fracture under normal usage loads.

#### 3.1.2. Limit Tolerances

The production of ceramic implants requires adherence to very strict specifications regarding the dimensions and uniformity of the components. If the dimensions do not meet the established tolerances, the implant may be prone to excessive local stresses when implanted. Even minor deviations from the specified tolerances can create zones of high mechanical stress that can weaken the implant and, over time, lead to its fracture.

### 3.2. Insertion

#### 3.2.1. Improper Insertion

The surgical process of implanting the liner into the body plays a crucial role in its longevity. Improper insertion can cause an unequal distribution of stresses across the surface of the ceramic implant. For instance, if the ceramic liner is not properly seated in the metallic component of the hip prosthesis, it may create a stress point that can initiate a crack in the material. Misalignment during surgery is a critical factor that can lead to premature failure of the implant.

#### 3.2.2. Limit Tolerances During Assembly with the Implant Head

**Figure 6:**
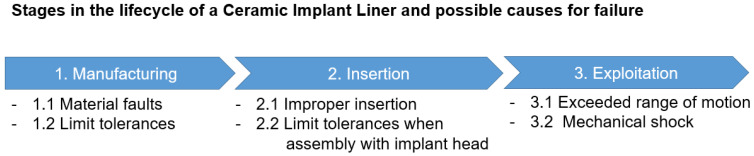
Stages in the lifecycle of a ceramic implant liner and possible causes of failure.

The assembly process between the ceramic liner and the femoral head of the implant must be precise to ensure proper functionality and long-term durability. If there are deviations in component dimensions or in the assembly method, additional stresses may be placed on the ceramic liner, increasing the risk of cracking or fracture under normal load during use. One illustration of this scenario is schematically presented in Figure 6.

**Figure 7:**
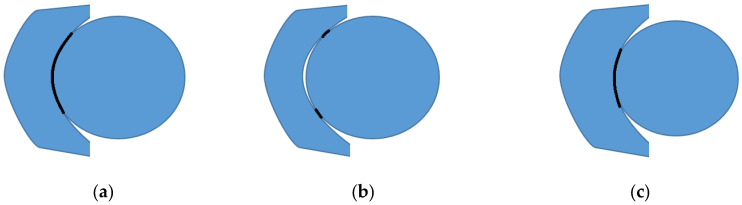
Schematics of liner–head assembly in a hip implant; (**a**) contact surface is large enough to dissipate optimally forces; (**b**,**c**) contact surface is small, inducing suboptimal force dissipation.

It is important to note that the schematics in Figure 7 are exaggerated, but the problem remains from a mechanical point of view due to the high rigidity of the CoC pair. From a mechanical point of view, the liner and head joint is a transition fit type of joint with equal nominal dimensions but with dimensional tolerances chosen to minimize the above presented effects.

### 3.3. Exploitation

#### 3.3.1. Exceeded Range of Motion or Impingement

**Figure 8:**
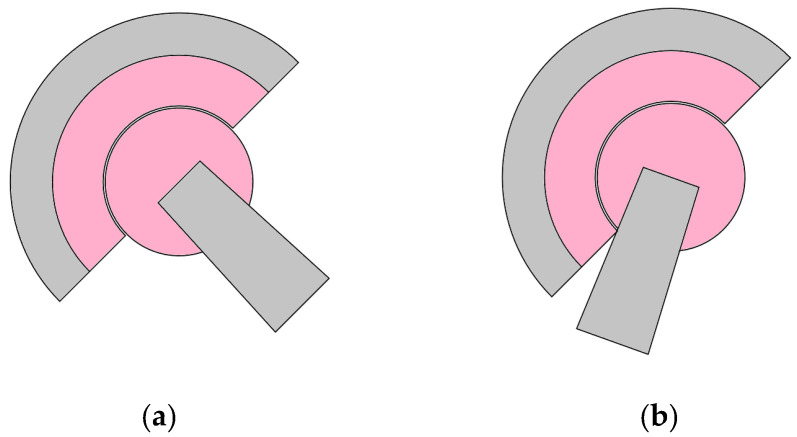
Femoral implant range of motion; (**a**) within limits; (**b**) impinging contact with the liner.

Ceramic is an extremely durable material but has specific limitations regarding elasticity and flexibility. If the patient performs extreme movements that exceed the normal range of the hip prosthesis, excessive mechanical forces may be applied to the ceramic liner, leading to failure. This risk is especially higher in active patients or those who do not follow post-operative instructions. A schematic of this situation is presented in Figure 8, where (a) represents the “in limits” position of the femoral implant head and neck, and (b) shows an impinging situation, where the implant neck collides with the liner, causing the ceramic to chip and cracks to be initiated.

#### 3.3.2. Mechanical Shock

Another major risk factor for ceramic implants is direct impact or mechanical trauma. For example, a fall or a strong blow to the hip area can cause a fracture of the ceramic liner. Even in the absence of a major impact, repeated mechanical shocks from daily activities, such as running on hard surfaces, can contribute to the wear and deterioration of the implant.

Also, one possible cause for implant failure can be corrosion due to fluids interacting with the implant or tribocorrosion due to friction between moving components at interfaces between contacting surfaces, as shown in [17]. This type of failure is clearly not present in the present case report; the present case report shows a fracture type of failure. An unusual and extreme case of tribocorrosion is presented in [18], where the implant head eroded even through the acetabular component, with the main conclusion being that the revision surgery would have been a less invasive procedure if failure had been detected earlier.

A study conducted in [19] shows that there is no obvious correlation for implant failure between the BMI—body mass index—and age, level of energy, and physical load; still, most patients in the study were classified as having a BMI in the obesity class I category. The patient presented in our study had a BMI of 22.9. In addition to the BMI, another risk factor is the age of the implant; this was shown in [20] to have a limited lifespan between 10 and 20 years, indicating that an implant received in younger patients leads to a higher risk for revision surgery.

## 4. Conclusions

In conclusion, while hip prosthesis implants have seen significant technological advancements, they are not immune to failure, which continues to present a substantial clinical concern. The case study of a catastrophic hip prosthesis liner fracture, leading to revision surgery and implant replacement, exemplifies the potential severity of these complications. By integrating this case with a broad review of the literature on liner failure mechanisms, we underline a range of contributing factors, from biomechanical stresses, material degradation, and implant design limitations to patient-specific variables such as anatomy, lifestyle, and underlying health conditions. We could not isolate a specific cause for the hip implant failure, but this situation is documented such that further analysis could be performed in larger studies. Taking into consideration the patient’s comorbidities, a study on the interaction between the zirconia-toughened alumina ceramic, used as liner and head, and the chemicals used for Hodgkin’s lymphoma treatment is suggested. However, the most probable cause for liner fracture is either a mechanical shock or impingement caused by an uncontrolled excessive motion. Although the patient did not remember a traumatic incident, an event as simple as a foot slipping on a surface can cause the implant to extend excessively and become damaged.

This underscores the multifaceted nature of hip implant success and failure, highlighting the importance of an interdisciplinary approach to ongoing research and clinical practice. The future of hip prosthetics hinges on advances in materials science, innovative engineering solutions, and the development of more robust implant designs that can withstand the complex mechanical forces exerted during everyday activities. Equally important is the refinement of surgical techniques and preoperative planning, incorporating personalized approaches tailored to each patient’s unique anatomy and activity levels.

Moreover, this case emphasizes the need for vigilant postoperative monitoring and the development of predictive tools to identify potential failure points before they lead to catastrophic outcomes. With an aging global population and increasing demand for joint replacements, the pressure on healthcare systems is growing. Continued collaboration between researchers, clinicians, and the industry will be essential to improve patient outcomes, extend implant longevity, and reduce the incidence of costly and invasive revision surgeries.

In a broader context, the lessons learned from this study reflect the importance of continuous innovation in medical technology and the role of personalized medicine in enhancing the quality of life for individuals requiring joint replacements. By addressing both the technological and clinical dimensions of implant performance, we can pave the way for more reliable and long-lasting solutions in the future.

## Data Availability

The original contributions presented in this study are included in the article. Further inquiries can be directed to the corresponding author.
